# Roles of PTEN with DNA Repair in Parkinson’s Disease

**DOI:** 10.3390/ijms17060954

**Published:** 2016-06-15

**Authors:** Mako Ogino, Mayuko Ichimura, Noriko Nakano, Akari Minami, Yasuko Kitagishi, Satoru Matsuda

**Affiliations:** Department of Food Science and Nutrition, Nara Women's University, Kita-Uoya Nishimachi, Nara 630-8506, Japan; turuchan22@gmail.com (M.O.); of16005@gmail.com (M.I.); okappi0214@yahoo.co.jp (N.N.); maiknaarmii@gmail.com (A.M.); y_kitagishi@live.jp (Y.K.)

**Keywords:** oxidative stress, ROS, DNA repair, PTEN, BRCA1, estrogen, Parkinson’s disease

## Abstract

Oxidative stress is considered to play key roles in aging and pathogenesis of many neurodegenerative diseases such as Parkinson’s disease, which could bring DNA damage by cells. The DNA damage may lead to the cell apoptosis, which could contribute to the degeneration of neuronal tissues. Recent evidence suggests that PTEN (phosphatase and tensin homolog on chromosome 10) may be involved in the pathophysiology of the neurodegenerative disorders. Since PTEN expression appears to be one dominant determinant of the neuronal cell death, PTEN should be a potential molecular target of novel therapeutic strategies against Parkinson’s disease. In addition, defects in DNA damage response and DNA repair are often associated with modulation of hormone signaling pathways. Especially, many observations imply a role for estrogen in a regulation of the DNA repair action. In the present review, we have attempted to summarize the function of DNA repair molecules at a viewpoint of the PTEN signaling pathway and the hormone related functional modulation of cells, providing a broad interpretation on the molecular mechanisms for treatment of Parkinson’s disease. Particular attention will be paid to the mechanisms proposed to explain the health effects of food ingredients against Parkinson’s disease related to reduce oxidative stress for an efficient therapeutic intervention.

## 1. Introduction

DNA damage and mitochondrial dysfunction may constitute a common pathway to neurodegenerative disorders [1,2]. However, initial underlying mechanisms that trigger the neurodegeneration in Parkinson’s disease (PD) are complex. PD is caused by the degeneration of dopaminergic neurons of the substantia nigra, which are required for proper motor function, and their loss is associated with tremor, bradykinesia, rigidity, and so on [3,4]. Dysfunction of mitochondria is thought to play an essential role in the neurodegeneration of the substantia nigra in brain, but the mechanisms involved remain unresolved. High levels of reactive oxygen species (ROS) are widely considered as a key event in the pathogenesis of PD, which is an intrinsic property of the vulnerable ventral midbrain dopaminergic neurons [5,6]. PD patients would worsen over time and suffer from considerable cognitive disability. To date, treatments are only symptomatic. Because mitochondria contain a number of enzymes essential for redox homeostasis, even a subtle defect in oxidative stress may cause not only fatal failure in neurons but also generates a large amount of ROS toxic to the vulnerable neurons in the central nervous system. Therefore, defective mitochondrial physiology may play a crucial role in the pathogenesis of PD. DNA damage and repair after the oxidative stress should activate the enzyme ataxia telangiectasia mutated (ATM) that is one of a cell cycle regulators [7,8]. Under stress conditions, those checkpoint proteins may play the key roles in genome protection by and mediating cellular response to DNA damage, and represent an important part of cellular stress response. A major focus of neuroscience research is to examine the precise mechanisms involved in the neuronal loss in order to find potential therapeutic targets [9,10,11]. Advances in a field of DNA damage and DNA repair biology have led to a better understanding of the molecular events significant in the pathogenesis of PD, and the understanding of the mitochondrial regulating pathways has raised several promising perspectives of neuroprotection. The knowledge of the mechanisms involved in the intracellular signaling cascades and molecular mechanisms which accomplish the process of DNA damage and DNA repair for neuroprotection could be critical for developing treatments to prevent and improve PD, other neurodegenerative disorders, and even aging to control the excess progression. 

## 2. DNA Damage and DNA Repair in Neurodegeneration

**Figure 1:**
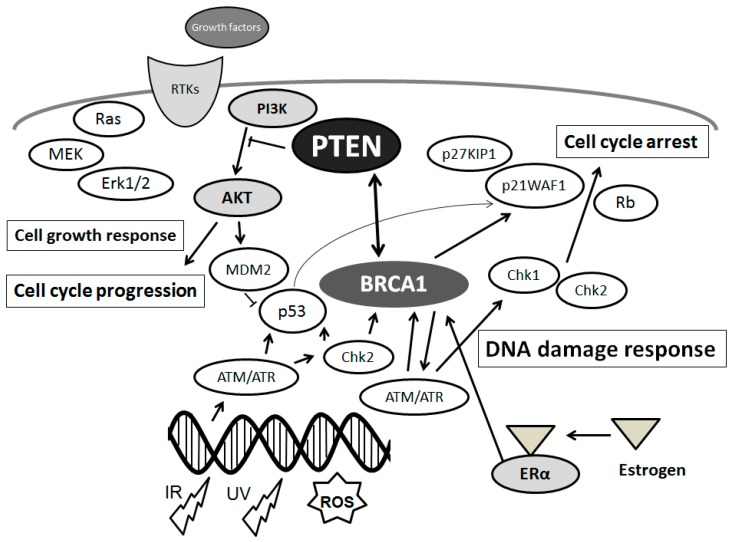
Schematic depiction of the integrative model of tumor suppressors signaling including *PTEN* and *BRCA1* and implication of estrogen action, DNA damage, and DNA repair systems. Typical molecules known to act on DNA damage response, cell proliferation, and cell cycle via the regulatory pathways are shown. Arrowhead means stimulation whereas hammerhead represents inhibition. Note that some critical pathways have been omitted for clarity.

**Figure 2:**
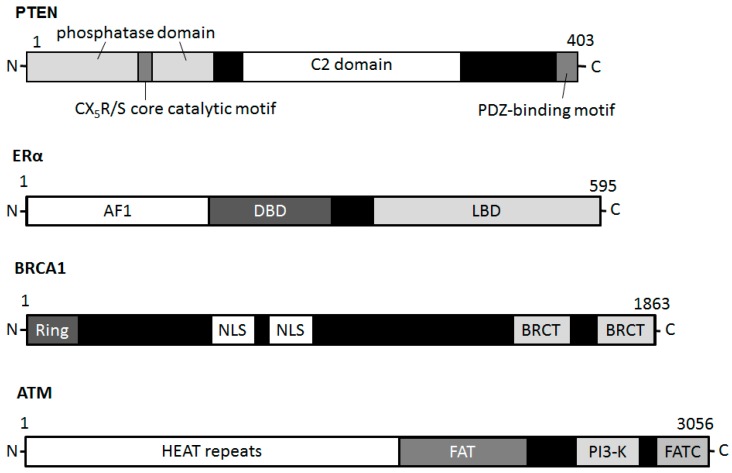
Schematic diagram indicating the domain structures of the PTEN, ERα, BRCA1, and ATM proteins. Note that the sizes of molecule are modified for clarity. PH domain = pleckstrin homology domain; C2 domain = a protein structural domain involved in targeting proteins to cell membranes; PDZ = a common structural domain in signaling proteins (PSD95, Dlg, ZO-1, *etc*.); AF1 = activation function 1; DBD = DNA-binding domain; LBD = ligand-binding domain; Ring = (Really Interesting New Gene) finger domain; NLS = Nuclear Localization Signal; BRCT = BRCA1 C Terminus; HEAT = huntington, elongation factor 3, a subunit of PP2A and TOR1; FAT = FRAP-ATM-TRRAP; FATC = FAT-C-terminal.

Oxidative damage is commonly involved in brain aging, neurodegeneration, and other neurological diseases, which can be created by usual cellular metabolism. Metabolic process produces ROS that accounts for oxidative damage to genome DNA [12,13]. In fact, increased oxidative DNA damage has been detected in nuclear and mitochondrial DNAs extracted from brain region of patients affected by neurodegenerative disorders [14,15,16]. Besides, neurodegenerative diseases are often associated with premature aging [17,18]. Oxidative DNA damage is one of the earliest detectable events in the neurodegeneration [19]. Convincing evidence has shown that oxidative DNA damage plays a central role in a programmed neuronal cell death, and is considered to be responsible for the degeneration of the dopaminergic neurons in PD [20]. However, machineries underlying the oxidative damage-mediated loss of the specific dopaminergic neurons in PD are not yet fully clarified. DNA damage is well-defined as a modification that changes its coding stuffs, and many different forms of DNA damage can be produced by both exogenous oxidative stressors and normal cellular functions. The process may involve a quantity of signaling cascades and molecules. High levels of mitochondrial genome mutations have been identified in dopaminergic neurons in the substantia nigra of neurodegenerative disease-patients relative to normal healthy controls [21,22], which has raised a speculation for an underlying connection between mitochondrial mutations and the neurodegeneration [21,22]. DNA damage is different from simple DNA mutations, which are alterations in base sequences of DNA. Generally, brain cells have an extraordinary level of metabolic activity and use discrete oxidative damage-repair mechanisms to remove the unacceptable many damages [23,24]. Hence, defective DNA repair system in brain cells could contribute to the neurological dysfunction. Standard cells have a machinery to maintain genomic integrity in response to the numerous genotoxic events. Under the genotoxic conditions, the cells do not move cell-cycle by activating the specific checkpoint in DNA damage [25,26,27,28] that acts as a procedure to transduce information from damaged DNA lesions to the cell-cycle regulator molecules. Then, DNA lesions can be repaired by DNA repair pathway. Initially, DNA damages accumulate with acute cellular stress conditions, which activate ATM, ataxia telangiectasia and Rad3 related (ATR), E2F, BRCA1, transcription factor and so on. In addition, pathways that have appeared as having crucial roles in both neurodegenerative diseases and cancer include those involving molecules such as ATM and PTEN [29] (Figure 1). Schematic structure of the predicted PTEN, ATM and BRCA1 is shown in Figure 2. Consequently, DNA damage induces G2/M arrest, which is a result from the activation of the ATM followed by the activation of Chk [30,31]. Aberrations of these molecules are frequently found in patients with neurodegenerative diseases [32]. In general, normal cells show a balance of the various mechanisms of the DNA damage and DNA repair machinery. Linking DNA damage and DNA repair with cell cycle inhibitors involving epigenetic regulatory phenomena might open the way to an attractive research topic in neurodegenerative diseases [16,33].

## 3. Association between PTEN and DNA Damage/DNA Repair Machinery in Neurodegenerative Diseases 

Phosphatase and tensin homolog on chromosome 10 (PTEN) is a dual-specificity phosphatase acting as a tumor suppressor, which has both protein phosphatase activity and lipid phosphatase activity that upsets PI3K activity [34,35]. The PI3K/AKT pathway transduces a signal regulating a wide range of events involved in cell survival and several functions (Figure 1). Cells without PTEN have constitutively upper levels of PIP3 and activate downstream PI3K/AKT targets [36,37]. Overexpression of PTEN may be closely related to the activation of the proteolytic cascade for apoptosis, which can be correlated with decreased activation of cell survival kinase AKT [38]. Accordingly, neuronal cell death may be attributed in part to the alterations in PTEN expression [39,40]. Regulation of apoptosis has been concerned in the pathogenesis of numerous neurodegenerative disorders. Therefore, it is important to identify neuronal pathways controlling apoptosis. Stimulation of AKT reduces oxidative stress levels and cell death, indicating that AKT activation by inactivation of PTEN is important to reserve its neuro-protective effect [41,42,43], which may construct a part of brain defense machinery against oxidative injury [41,42,43]. It has been shown PTEN insufficiency results in the increase of several mitochondrial activities, along with an activation of the PI3K/AKT signaling pathway, of which PTEN may be a negative regulator [44]. In addition, a ubiquitously expressed molecule PTEN-induced kinase 1 (PINK1) has been shown to have a physiological role in mitochondrial conservation, suppressing mitochondrial oxidative stress, oxidative DNA damage, and autophagy [45,46]. PINK1 knockdown increases the neuronal apoptosis, whereas PINK1 overexpression returns it [47,48,49]. PINK1 plays crucial roles in the regulation of mitochondrial function and dynamics, and mutations in PINK1 have been linked to genetic forms of PD [50]. Protective role of PINK1 in neuronal cells against oxidative stress has also been suggested for developing novel strategy to the treatment of neurodegenerative diseases. It has been shown that NADPH oxidase-mediated ROS production elicits oxidation and inactivation of PTEN resulting in upregulation of the signaling [51]. Similarly, PTEN is oxidized and inactivated by the acidosis-induced ROS [52]. Inhibition of PTEN by ROS is needed for recruiting downstream signaling molecules such as AKT in insulin-mediated signaling [53]. The major form of oxidative DNA damage base in the DNA is 8-oxo-7,8-dihydro-2′-deoxyguanosine (8-oxodG). Arsenite-induced oxidation increases the 8-oxodG and decreases PTEN levels [54]. However, how PINK1 is involved in the neuronal cell survival against oxidative stress and damage remains not precisely clarified.

As mentioned above, main DNA damage recognition molecule is ATM, which is a cell-cycle checkpoint kinase that phosphorylates a number of proteins including p53 and BRCA1 in response to DNA damage (Figure 2), and thus induces the response to DNA damage [32,55]. PTEN plays a role in G2/M arrest by facilitating the activation of the ATM (Figure 2) pathway required for the proper activation of checkpoints in response to DNA damages. In addition, PTEN has serious roles in the regulation of genomic instability and DNA repair pathways [56,57]. Knockdown of PTEN strongly antagonizes the ATM activation and thereby reduces the phosphorylation level of ATM-substrates including Chk, H2AX, and p53 [58,59]. Recent studies have shown that PTEN plays distinctive roles in the DNA damage response and can interact with the Chk pathway [59]. Formation of phospho-H2AX is an integral part of the signaling pathway for double strand break repairs in which PTEN may be involved [60,61]. Genomic instability eventually induces cell death and/or apoptosis [62]. However, little is known about how PTEN contributes to DNA damage responses. BRCA1 is a well-known breast cancer tumor suppressor, which is to associate with breast cancer risk and genetic susceptibility [63]. Several studies have revealed that BRCA1as well as PTEN plays a critical role in DNA damage responses. The PI3K/AKT pathway is constitutively active in BRCA1-defective cells [64]. PTEN has been shown to trans-activate RAD51 promoter with a member of the E2F transcription molecule family [65]. Since the E2F complexes associate with histone modifying enzymes, histone modifications together with its acetylation and methylation are required for cell cycle regulation in DNA repair system. Furthermore, PTEN is a target of nitric oxide (NO) and/or hydrogen peroxide, and the oxidative modification of cysteine residue diminishes its enzymatic activity [66]. Phosphatase activity of PTEN is inhibited via the transnitrosylation reaction such as a transfer of NO from the cysteine residue of a protein to another site [67]. PTEN has been shown sensitive to NO under the conditions. Detoxification of ROS and/or reduction of ROS could protect neurons. Importantly, nitrosylation of PTEN decreases the phosphatase activity of PTEN, thus in consequence activating PI3K/AKT, and then promoting cell survival.

## 4. Estrogen and Its Receptor Signaling with PTEN Involved in PD

Numbers of genetic researches suggest that the pathogenesis of PD and cancer may include similar mechanisms and pathways [68]. Actually, PTEN is a famous tumor suppressor that is functionally associated with numerous human cancers. *PTEN* and *BRCA1* are recognized as often deleted and/or mutated genes in several human cancers. The BRCA1 molecule functionally cooperates with PTEN and might be an indispensable blockage against the development of several tumors [36]. PD is initiated by the specific degeneration of dopaminergic neurons, whereas cancer results from the immoral growth of malignant cells. Differences in the pathological and intracellular signaling mechanisms may result in two such divergent diseases. Recent studies have informed a relationship between DNA repair deficiency and loss of sex hormone signaling [69,70]. Estrogens are primary female sex hormones and play important roles in the reproductive systems, which can be synthesized even in non-reproductive tissues such as heart, liver, brain, muscle, and bone. Estrogens not only show a fundamental role in cell proliferation and differentiation on several epitheliums but also are involved in control diverse cellular processes. Tissue-specific synthesis may be consistent with a variety of the estrogen actions. Interestingly, studies have shown that mitochondria are an imperative target of estrogen [71,72]. Estrogen augments mitochondrial role by enhancing mitochondrial biogenesis and supporting mitochondrial capacity. Hence, estrogen has been suggested as a possible therapeutic mediator against neurodegeneration due to the strong anti-oxidant and anti-apoptotic properties. Estrogens also induce the expression of genes such as BRCA1 that can act to repair the DNA damage. Contemporary studies have shown an association between DNA repair deficiency and loss of specific estrogen receptors (ERs) [16] (Figure 2). In addition, two major ERs-dependent signaling seems to be involved in the neuroprotective roles against PD [73,74]. In PD experimental animal model, estrogen-treatment protects against neuronal cell death, and an inhibitor of the PI3K/AKT pathway blocks the survival effects of estrogen to dopamine neurons. Estrogen may mediate neuroprotection through the inactivation of PTEN activity and/or decrease in PTEN expression level [75,76], which may be used as a key parameter for evaluation of the efficacy of estrogen in prevention of neuronal degeneration. In contrast, estrogen-related receptor α is up-regulated in the absence of PTEN [77]. PI3K/AKT activity also regulates the expression of estrogen-related receptor α and mitochondrial respiration [77]. Therefore, estrogen may be an effective novel treatment approach to inhibit the neuronal cell death. Foods such as soy and beans are sources of phyto-estrogens. The phyto-estrogens protect cells against ROS by scavenging free radicals. In addition, these hormones have direct effects on differential mediators of the mitochondriogenesis procedure [78].

**Figure 3:**
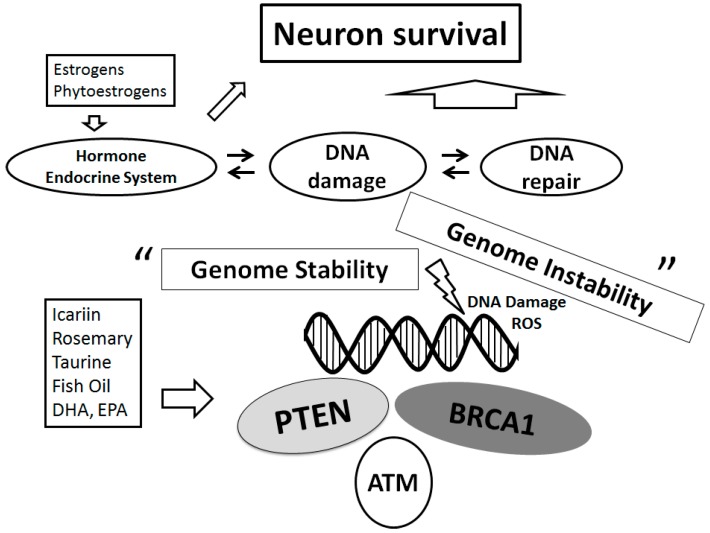
Schematic representation of the DNA repair system for neuroprotection implying that genome stability is sustained on several tumor suppressors. Examples of the action known to act on the neuron survival pathways are shown. Several dietary modulators linked to this pathway are also demonstrated, whose potential molecular targets may be based on the predominant sites. Note that some critical functions have been omitted for clarity.

Mitochondrial dysfunction has been reported in most neurodegenerative diseases. Specific deletion of PTEN and/or estrogen receptors is associated with high oxidative stress, increased mitochondrial form and augmented respiration [77]. In addition to the specific deletions, some other mechanisms might change the activities of PTEN and/or estrogen receptors. For example, PTEN inactivation is caused by somatic mutations, inherited germline mutations, epigenetic silencing, post-translational modifications, and molecular interactions [79]. Epigenetic mechanisms such as histone modifications, DNA methylation, and small RNA-mediated mechanisms, could regulate the expression of PD-related genes including PTEN [80]. Accordingly, linking DNA damage and DNA repair with epigenetic phenomena opens the way to an attractive research topic in neurodegenerative diseases [16]. A low PTEN expression due to the methylation may also contribute to malignant cancer progression and could be useful for the prognosis [81]. Particular attention should be paid to the molecular mechanisms suggested to explain the cellular wellbeing-effects such as neuroprotection against the diseases related to oxidative stress driven pathologies including neurodegenerative disease and/or PD (Figure 3). Indeed, the knowledge of mechanisms involved in this cellular protection could be critical for developing treatments to prevent the neurodegenerative disorders to control its excess progression.

## 5. Diets May Contribute to the Improved Neuron-Survival via the Modulation of PI3K/AKT/PTEN Signaling

Effective therapeutic strategies should exploit the remark that defects in significant processes required for cellular homeostasis, which produce an alternative functional situation (Figure 3). Dietary interventions to reduce PTEN expression could contribute to the prevention of the neurodegenerative diseases and/or decrease the speed of its progress. Lately, omega-3 (n-3) long-chain polyunsaturated fatty acids (PUFAs) have become an attention of interest in neurology [82]. Particularly, docosahexaenonic acids (DHA) are important for brain development and neuronal health [83]. Consequently, fish oil administration may retain hippocampal neurons and recovers cognitive deficit by activation of PI3K/AKT signaling [84]. Fish oil diet modifies the level of PTEN protein [85]. In this way, neuroprotection could be performed by certain diets involved in the PI3K/AKT/PTEN pathway. Curcumin, an active ingredient derived from the root of the plant *Curcuma longa* widely used as culinary spice turmeric, can improve plasticity of synapse, which then enhances memory abilities [86,87]. It is proposed that the neuroprotection of curcumin might be facilitated via the alteration of PI3K/AKT activity [88,89]. Several plants or fruits may also be favorable. Kaempferol is a flavonol that is present in various plants including some edible berries and grapefruits, which has neuroprotective effects in the mouse model of PD [90]. In addition, some component of rosemary herb inhibits the PTEN expression in K562 myeloid cell line cells [91]. Icariin is a neuroprotective ingredient of *Herba Epimedii*, a traditional Chinese medicinal herb, which also inhibits the PTEN expression following AKT activation [92,93,94]. Taurine, a sulfur-containing amino acid located in mammalian tissues in high concentrations, possesses a variety of biological actions, such as ion movement, antioxidant capacity, osmoregulation and mitochondrial function [95]. AKT and PTEN phosphorylation is significantly increased by taurine supplementation, resulting PTEN inactivation and AKT activation [96]. In addition, a high-fat diet increases circulating fatty acids, which considerably modifies the PTEN expression [97].

On the contrary, Honokiol, an active component isolated and purified from Chinese traditional herb magnolia, may be able to decrease the PI3K/AKT signaling by upregulation of the PTEN expression [98]. Soy isoflavones at physiologically relevant concentrations by dietary exposure also stimulates PTEN expression [99]. In addition, genistein, a major isoflavone found in soybeans upregulates PTEN expression [100]. Both quercetin and genistein have an influence to up-regulate PTEN transcription, then, suppresses the PI3K/AKT signaling pathway. Generally, phytoestrogen exposure may cause a transcriptional increase in PTEN expression. Biological activity of the isothiocyanates, rich in certain vegetables such as broccoli, has been demonstrated to suppress AKT signaling [101]. Indole-3-carbinol, a phytochemical found in certain vegetables, upregulates PTEN in experimental animal model [102,103]. Interestingly, animals with tryptophan-restriction show a reduced activity of phosphorylated AKT [104]. However, in spite of these experimental interpretations, the accurate mechanisms for those food ingredients remain obscure for clinical practices. Furthermore, it seems imperative to exploit the potential profits of optimal treatment and/or combination with chemical modulators on PI3K/AKT pathway.

## 6. Perspective

There is an imperative requisite to recognize more about DNA repair pathways. The DNA repair system is an extremely conserved DNA excision process that keeps genomic fidelity through the credit and repair of the damaged nucleotides. Because apoptotic cell death is demonstrative of neurodegeneration, down-regulation of PTEN may be required to prevent the progression of neurodegenerative disorders. In addition, signaling pathway of several hormones has a complex network, and thus further comprehensive research in this area is mandatory for investigation. Detection of the other molecular pathways would be supportive to better understand the essential mechanisms of hormone dependence to the neuroprotection. It is plausible that certain food ingredients including phytoestroten act as antioxidants by scavenging ROS to protect certain neurons from DNA damage via the modulation of PI3K/AKT pathway. Further systematic studies are needed in order to realize the precise molecular mechanisms of hormonal and PTEN involved DNA repair scheme for effective prevention and therapeutic intrusions of PD.

## Abbreviations

ATM	ataxia telangiectasia mutated
ATR	ataxia telangiectasia and Rad3 related
BRCA1	breast cancer susceptibility gene 1
DHA	docosahexaenoic acid
ER	estrogen receptor
n-3 PUFA	omega-3 polyunsaturated fatty acid
NO	nitric oxide
8-oxodG	8-oxo-7,8-dihydro-2′-deoxyguanosine
PD	Parkinson’s disease
PI3K	Phosphoinositide 3-kinase
PINK1	PTEN-induced kinase 1
PTEN	phosphatase and tensin homolog on chromosome 10
PUFAs	polyunsaturated fatty acids
ROS	reactive oxygen species
